# Effects of the reaching married adolescents program on modern contraceptive use and intimate partner violence: results of a cluster randomized controlled trial among married adolescent girls and their husbands in Dosso, Niger

**DOI:** 10.1186/s12978-023-01609-9

**Published:** 2023-06-05

**Authors:** Jay G. Silverman, Mohamad I. Brooks, Sani Aliou, Nicole E. Johns, Sneha Challa, Abdoul Moumouni Nouhou, Shweta Tomar, Holly Baker, Sabrina C. Boyce, Lotus McDougal, Stephanie DeLong, Anita Raj

**Affiliations:** 1 Center On Gender Equity and Health, School of Medicine, University of California San Diego, La Jolla, CA 92093 USA; 2 Pathfinder International, Watertown, USA; 3 GRADE Africa-Niger, Niamey, Niger

**Keywords:** Niger, Family planning, Contraception, RCT, IPV, Behavior change, Adolescent marriage, Adolescent fertility

## Abstract

**Background:**

Niger has the highest rate of adolescent fertility in the world, with early marriage, early childbearing and high gender inequity. This study assesses the impact of Reaching Married Adolescents (RMA), a gender-synchronized social behavioral intervention designed to improve modern contraceptive use and reduce intimate partner violence (IPV) among married adolescent couples in rural Niger.

**Methods:**

We conducted a four-armed cluster-randomized trial in 48 villages across three districts in Dosso region, Niger. Married adolescent girls (ages 13–19) and their husbands were recruited within selected villages. Intervention arms included home visits by gender-matched community health workers (CHWs) (Arm 1), gender-segregated, group discussion sessions (Arm 2), and both approaches (Arm 3). We used multilevel mixed-effects Poisson regression models to assess intervention effects for our primary outcome, current modern contraceptive use, and our secondary outcome, past year IPV.

**Results:**

Baseline and 24-month follow-up data were collected April–June 2016 and April–June 2018. At baseline, 1072 adolescent wives were interviewed (88% participation), with 90% retention at follow-up; 1080 husbands were interviewed (88% participation), with 72% retention at follow-up. Adolescent wives had higher likelihood of modern contraceptive use at follow-up relative to controls in Arm 1 (aIRR 3.65, 95% CI 1.41–8.78) and Arm 3 (aIRR 2.99, 95% CI 1.68–5.32); no Arm 2 effects were observed. Relative to those in the control arm, Arm 2 and Arm 3 participants were significantly less likely to report past year IPV (aIRR 0.40, 95% CI 0.18–0.88 for Arm 2; aIRR 0.46, 95% CI 0.21–1.01 for Arm 3). No Arm 1 effects were observed.

**Conclusions:**

The RMA approach blending home visits by CHWs and gender-segregated group discussion sessions is the optimal format for increasing modern contraceptive use and decreasing IPV among married adolescents in Niger.

*Trial registration* This trial is retrospectively registered with ClinicalTrials.gov, Identifier NCT03226730

**Supplementary Information:**

The online version contains supplementary material available at 10.1186/s12978-023-01609-9.

## Plain language summary

Although Niger has both the highest levels of fertility and of child marriage in the world, as well as substantial gender inequity, there have been no high-quality evaluations of public health programs aiming to increase contraceptive use or decrease intimate partner violence. In this study, we conducted a high quality, randomized controlled trial to evaluate whether the Reaching Married Adolescents public health program could increase modern contraceptive use and decrease intimate partner violence among married adolescent girls (13–19 years old) and their husbands in the Dosso region of Niger. The results of this evaluation provide evidence of the value of individual home visits for wives and their husbands in increasing modern contraceptive use, the value of small group discussions in reducing intimate partner violence, and the combined value of receiving both approaches at the same time for both increasing modern contraceptive use and decreasing intimate partner violence. The current study advances the state of evidence regarding contraceptive use and IPV among married adolescents and their husbands in Niger, highlighting the importance of engaging male partners in such public health programs, as well as of using multiple modes of delivery of programs. The success of this intervention in the high-risk context of Niger suggests that other countries in the region may benefit from testing this approach to improve the health and well-being of young wives.

**Supplementary Information:**

The online version contains supplementary material available at 10.1186/s12978-023-01609-9.

## Background

The West African Francophone country of Niger has the highest prevalence of girl child marriage (marriage < 18 years) in the world [1, 2]. The median age at marriage for girls is under 16 years, with 1 in 4 married by age 15, and 3 in 4 married by age 18 [2]. Early marriage has been linked to multiple adverse health outcomes (e.g., maternal and infant mortality), many of which stem from early childbearing [3, 4]. Social norms support early childbearing in Niger [5], with a median age at first birth of 18.2 for rural-residing women [1]. This early childbearing is experienced in tandem with low contraceptive prevalence, with fewer than 6% of married 15–19 year old girls using modern contraception, contributing to the highest levels of fertility in the world for adolescent Nigerien girls [1, 5, 6].

Across sub-Saharan Africa, adolescent girls face myriad structural, community, and interpersonal barriers to contraceptive use [7]. While there is need for increased health and contraceptive service access, service delivery, and health worker training at the structural level in Niger, ethnographic work has shown that social norms regarding gender roles have had a profound impact on adolescent girls’ reproductive health [8]. Niger experiences pervasive gender inequalities, as reflected in its nearly last-place ranking (154 of 162 countries) on the 2018 Gender Inequality Index [9]. At the community level, these gender norms set expectations that men are providers and heads of household while women’s primary responsibility is to bear and raise as many children as is feasible, precluding their use of contraception [10].

At the interpersonal level, power dynamics favor husbands’ control over both household- and fertility-related decisions, with only 7% of married women in Niger made their own decisions regarding sex, contraception, and healthcare [10, 11]. The number of children a woman has is a major determinant of her social status in rural Nigerien communities, greatly limiting nulliparous married adolescent girls’ access to social resources and decision-making power [10, 12]. This prescribed male dominance is often maintained, in part, via intimate partner violence (IPV), which is consistently associated with traditional gender role beliefs and low female educational, economic, and social status in sub-Saharan Africa [13]. Ultimately, these experiences of violence likely limit young women’s access to, and control over, contraceptives, perpetuating high unmet need for contraception and subsequent unintended and poorly spaced pregnancies [14–16]. Recent studies in Niger support this contention, with IPV linked to contraceptive use only in cases where that use was not known to husbands (i.e., covert use), indicating women’s and girls’ resistance to restrictive gender norms and pursuit of reproductive autonomy [17].

There is critical need for family planning programs in Niger, to provide quality sexual and reproductive health (SRH) services for married adolescents, but also to address the gendered power dynamics that compromise reproductive agency for married girls in this context. Based on this premise, Pathfinder International developed the Reaching Married Adolescents (RMA) program to promote modern contraceptive use, and greater female control thereover, by increasing knowledge, attitudes, and norms supportive of modern contraceptive access and female reproductive autonomy [18]. RMA engaged adolescent wives and their husbands in household visits and small group discussions, and simultaneously engaged village members, including religious leaders, in community dialogues. This model was informed by previous research demonstrating the efficacy of interventions that utilized household visits to increase acceptance of contraceptive use in India (the PRACHAR study [19]), the utility of male engagement in promoting reproductive health decision-making discussions in Niger (Husbands’ Schools [20]) and those utilizing single-sex, small group sessions among wives and husbands to increase gender norms supportive of female decision-making related to sexual and reproductive health in Uganda (the GREAT study [21]).

A cluster randomized controlled trial (cRCT) was conducted to evaluate the effects of the RMA program on current modern contraceptive use (primary outcome) and past year experiences of physical and sexual IPV (secondary outcome) [18]. This cRCT is one of the first experimental efforts in Niger to include a population-based sample of married adolescent wives and their husbands. The four-armed design allows for robust assessment of the relative efficacy of commonly-implemented intervention modalities, and offers much-needed evidence on the effects of these approaches on contraceptive use and gender equity in rural West Africa.

## Methods

### Study design and participants

We evaluated the effects of the RMA intervention using a factorial, 4-arm cRCT, including a control arm and three intervention arms: household visits only (Arm 1), small group sessions only (Arm 2), and both household visits and small group sessions (Arm 3); all intervention arms included community dialogues. Full study protocol is available elsewhere [18].

We recruited participants from 48 villages selected from the Dosso, Doutchi, and Loga districts in the Dosso region of Niger. Eligibility criteria for villages included (1) having at least 1000 residents; (2) being primarily Hausa or Zarma-speaking (the two major languages of central southern Niger); and (3) having received no intervention specific to contraceptive use or gender equity. Eligibility criteria for participants within selected villages included (1) being Hausa or Zarma speaking; (2) not planning to move away from the village in the next 18 months; (3) not planning to travel away from the village for more than 3 months during the next 18 months; and (4) not being sterilized.

### Randomisation

The three eligible districts in the Dosso region were randomly assigned to an intervention condition using a computer-generated random number list. Within each district, we used a different computer-generated random number list to select 16 villages among those meeting inclusion criteria; four of those 16 villages in each district were randomly assigned to the control condition, for a total of 12 villages in each of the four study conditions (control, Arm 1, Arm 2, Arm 3). Within each of the selected villages, we randomly selected 25 households inclusive of a married female adolescent aged 13–19 years and her husband using from a list of all such households generated with the assistance of the village chief. Research assistants visited the randomly selected households to confirm eligibility; those not meeting these criteria were replaced by a household randomly selected from those remaining of the list.

### Procedures

RMA is a community-based, gender-synchronized program implemented by Pathfinder International and designed to increase use of modern spacing contraception among married adolescent girls and their husbands. Based on the Theory of Reasoned Action [18], the model includes household visits to individual wives and husbands, single-sex small discussion groups, and village-level community dialogues intended to increase modern contraceptive knowledge, use, and supportive attitudes and norms. RMA also promoted gender equitable attitudes and norms, particularly the role of women and girls in contraceptive decision-making. Additional details of the intervention, sample, and trial design are available in the protocol paper [18].

Gender-matched, trained community health workers (CHWs) conducted household visits to married adolescent girls and their husbands. Household visits to married adolescent girls included monthly visits providing information and counseling on healthy timing and spacing of pregnancies and how to access and use modern contraceptive methods. Monthly household visits to husbands included these same topics. Selected female and male community members served as “mentors” and were trained to facilitate small, single-sex groups for married adolescent girls (twice monthly) and their husbands (once monthly). Content delivered in these groups included general health and life skills, reproductive health and anatomy, use of modern contraceptive methods for healthy timing and spacing of pregnancies, gender norms that impede contraceptive use and female autonomy, couple communication regarding fertility decisions, and gender-based violence. Community dialogues were convened by two trained facilitators each month at the village-level, engaging community gatekeepers and key influencers (e.g., religious and community leaders, parents, and in-laws) to create an environment supportive of healthy timing and spacing of pregnancies, including modern contraceptive use among married adolescent girls and their husbands.

Data for the current study were collected via in-person surveys administered at two time points: April through June 2016 (baseline; T1) and April through June 2018 (24-month follow-up). Surveys were conducted verbally with participating wives and husbands (separately) in locations deemed to provide audio privacy. Gender-matched, trained research assistants obtained verbal consent from study participants prior to survey administration; questions were administered using tablet computers in either Hausa or Zarma and required 40–60 min to complete. Protocols incorporated World Health Organization guidelines for conducting research on violence against women^5^ to protect the safety and confidentiality of women and girls participating in the study.

Patient privacy and confidentiality was maintained through de-identified data collection, separate encrypted file storage of any personally identifiable information collected, daily survey data backup, and sharing/analysis of only de-identified data [18]. Enumerators were trained to monitor for adverse events during intervention delivery and data collection, and to report adverse events to study administrators in Niger and at the University of California San Diego.

Ethics review boards of the University of California San Diego and the Niger Ministry of Health approved all study procedures (see protocol [18] for additional details).

### Outcomes

The primary outcome of this study was current use of modern contraceptives among female study participants. To assess this use, women were first asked whether they had ever done anything to delay or limit their number of pregnancies. Those answering in the affirmative were then asked about whether they had ever used each of the following methods: intrauterine device (IUD), injectable, implant, contraceptive pill, male condom, female condom, emergency contraception, and lactational amenorrhea (LAM). Those answering yes to having ever used any of these modern methods were then asked if they were using this method currently. Current use of modern contraceptives was defined as a response of ‘yes’ to any question about current use of any of these methods. All analyses of current contraceptive use excluded women who were currently pregnant.

The secondary outcome of this study was past year intimate partner violence (IPV) among adolescent wives. Eight items from the Demographic and Health Surveys domestic violence module were utilized to assess adolescent wives’ experiences of physical and/or sexual IPV during the prior 12 months. Female participants were asked whether their current husband had ever: (a) pushed her, shaken her, or thrown something at her; (b) slapped her; (c) twisted her arm or pulled her hair; (d) hit her with his fist or something that could hurt her; (e) kicked her, dragged her, or beat her up; or (f) choked her or tried to burn her. They were also asked whether their husband had physically forced them to (a) have sexual intercourse when they did not want to, or (b) to perform any other sexual acts they did not want to. If a participant answered ‘yes’ to an item, they were asked whether this behavior has occurred in the past 12 months. Past year IPV was defined as a response of ‘yes’ to one or more of the questions regarding occurrences in past 12 months. Additional secondary outcomes of this study pertaining to contraceptive knowledge, attitudes, community norms, self-efficacy and intentions [18] are being analyzed separately.

### Statistical analysis

Power calculations were conducted a priori based on the primary study outcome, modern contraceptive use. We assumed an intra-cluster correlation coefficient of kappa = 0.05. To provide 80% power at capturing an effect size of 2.0 greater odds of modern contraceptive use across four arms with 12 clusters of equal size nested within each arm, with 10% anticipated attrition, and 95% confidence, we calculated that 300 couples would be required at baseline (1200 wives and their husbands) [18].

Changes between baseline and follow-up comparing intervention arms to the control condition were assessed with an intention to treat, difference-in-difference Poisson regression approach, using mixed-effects models with nested random effects accounting for village-level clustering. Minimally adjusted models accounted only for time (baseline or follow-up), treatment status or study arm, district, and a time-treatment interaction term. Such models were constructed both considering combined treatment status (i.e. control vs. pooled intervention arms) and specific study arm (i.e. control vs. Arm 1 vs. Arm 2 vs. Arm 3). Fully adjusted models included baseline demographic characteristics as fixed effects in outcome models if they were associated with treatment or female loss to follow-up in Fisher’s exact tests or ANOVA tests at p < 0.20; female loss to follow-up was used because female data defined outcome measures. Fixed effects in these models thus included baseline values of wife age at marriage, wife education, husband education, wife parity, husband polygamy, husband migration for more than 3 months of the previous year, and household asset ownership (watch, mobile phone, bicycle, motor bike/scooter, car/truck, animal-drawn cart). Wife’s age was included based on documented associations and programmatic interest [17]. Both unadjusted and adjusted models included individuals nested within villages as random intercepts. All models utilized robust variance estimation specification. We examined but did not find evidence of collinearity, with all included variables having variance inflation factor < 2.

Based on the importance of women’s age and parity in reproductive autonomy and IPV [5, 7, 14], as well as prior associations in this population [17], we also conducted baseline age- and baseline parity-stratified post-hoc analyses for primary and secondary outcome; these models were minimally adjusted as a result of small cell sizes. We conducted an additional post-hoc sensitivity analysis utilizing inverse probability of censoring (IPC) weighting to account for the influence of greater loss to follow-up in some groups. Weights were based on socio-demographic characteristics associated with female loss to follow-up at p < 0.20 (wife age at marriage, wife parity, husband polygamy, and husband migration for more than 3 months of the previous year) based on primary and secondary outcomes. Weights were truncated at the 95th percentile and missing weights were replaced with median weight value. These IPC weights were used in difference-in-difference Poisson regression models, controlling for time, study arm, time-by-study arm interaction (to assess treatment effect), and district, and including nested random intercepts of individual within village.

We conducted all analyses using STATA 15.1. Significance was set at p < 0.05 for all Fisher’s exact tests, ANOVA tests, and incident rate ratios (IRRs); 95% confidence intervals (CIs) are reported throughout. This trial is registered with ClinicalTrials.gov, Identifier NCT03226730.

### Role of the funding source

The funder of the study had no role in study design, data collection, data analysis, data interpretation, or writing of the report.

## Results

**Fig. 1:**
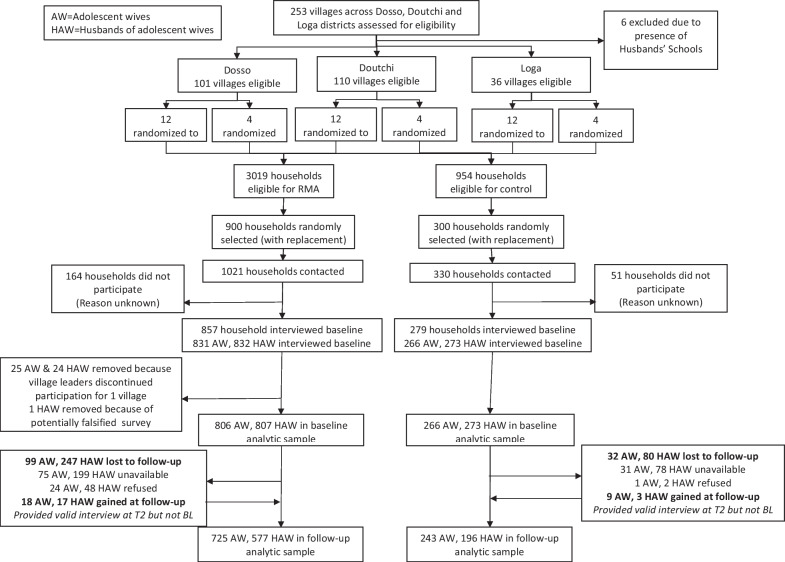
RMA trial profile

Participants were recruited between April and June 2016 for baseline (T1) interviews, and contacted again between April and June 2018 for 24-month follow-up interviews. At baseline, surveys were collected from 1072 of 1351 eligible adolescent wives (79.3% female participation), 968 of whom provided survey data at follow-up (90.3% female retention); 1080 of 1351 eligible husbands completed surveys at baseline (79.9% male participation), of whom 773 participated in data collection at follow-up (71.6% male retention) (Fig. 1). Additionally, 27 women who did not provide surveys at baseline but provided surveys at follow-up were added to the sample. In total, 1099 women were included in the final sample. Intervention delivery and 24-month follow-up data collection were completed as outlined in the protocol paper [18].

Average age of wives at baseline was 17.3 years [standard deviation (SD) 1.5 years], and average age of husbands at baseline was 25.6 years (SD 5.3 years) (Table 1). Wives were, on average, 14.2 years old at marriage (SD 1.9 years) (Table 1). Education was low for both women and men, with limited levels of Quranic school (16% women, 20% men) and any modern school (35% women, 47% men), and high levels of no schooling (48% women, 30% men). At baseline, 40% of adolescent wives had never given birth, and most husbands (84%) had only one wife. Few wives had travelled outside the village for more than 3 months in the past year (6%), but this was common for husbands (67%). Of six assets assessed in the baseline survey, households owned on average 2.1 (SD 1.2 assets).

**Table 1:** Baseline characteristics of individual participants

	Overall	Treatment status		Intervention arms		
	Total^a^	Control	Intervention (Arms 1–3 combined)	Arm 1—home visits only	Arm 2—group sessions only	Arm 3—home visits and group sessions
N (adolescent wives)	1099	275	824	289	261	274
Wife age (years), mean (SD)	17.3 (1.5)	17.4 (1.5)	17.3 (1.6)	17.4 (1.5)	17.0 (1.7)	17.4 (1.5)
Wife age at marriage (years), mean (SD)	14.2 (1.9)	14.3 (2.0)	14.1 (1.9)	14.2 (1.7)	13.8 (2.0)	14.3 (1.9)
Husband age (years), mean (SD)	25.6 (5.3)	25.4 (5.4)	25.6 (5.3)	25.6 (5.3)	26.4 (5.5)	24.9 (5.0)
Couple age difference (Husband–Wife), mean (SD)	8.3 (5.0)	8.0 (5.1)	8.4 (5.0)	8.2 (4.9)	9.4 (5.2)	7.5 (4.7)
Wife education						
Any modern	386 (35.1%)	84 (30.5%)	302 (36.7%)	92 (31.8%)	107 (41.0%)	103 (37.6%)
Quranic only	178 (16.2%)	54 (19.6%)	124 (15.0%)	7 (2.4%)	86 (33.0%)	31 (11.3%)
No schooling	524 (47.7%)	134 (48.7%)	390 (47.3%)	188 (65.1%)	66 (25.3%)	136 (49.6%)
Missing	11 (1.0%)	3 (1.1%)	8 (1.0%)	2 (0.7%)	2 (0.8%)	4 (1.5%)
Husband education						
Any modern	517 (47.0%)	110 (40.0%)	407 (49.4%)	148 (51.2%)	133 (51.0%)	126 (46.0%)
Quranic only	219 (19.9%)	63 (22.9%)	156 (18.9%)	25 (8.7%)	78 (29.9%)	53 (19.3%)
No schooling	325 (29.6%)	94 (34.2%)	231 (28.0%)	108 (37.4%)	40 (15.3%)	83 (30.3%)
Missing	38 (3.5%)	8 (2.9%)	30 (3.6%)	8 (2.8%)	10 (3.8%)	12 (4.4%)
Wife parity						
None	438 (39.9%)	111 (40.4%)	327 (39.7%)	112 (38.8%)	101 (38.7%)	114 (41.6%)
1 birth	367 (33.4%)	95 (34.5%)	272 (33.0%)	91 (31.5%)	99 (37.9%)	82 (29.9%)
2 or more births	287 (26.1%)	68 (24.7%)	219 (26.6%)	85 (29.4%)	59 (22.6%)	75 (27.4%)
Missing	7 (0.6%)	1 (0.4%)	6 (0.7%)	1 (0.3%)	2 (0.8%)	3 (1.1%)
Husband monogamous or polygamous						
Monogamous	922 (83.9%)	239 (86.9%)	683 (82.9%)	232 (80.3%)	219 (83.9%)	232 (84.7%)
Polygamous	145 (13.2%)	29 (10.5%)	116 (14.1%)	49 (17.0%)	33 (12.6%)	34 (12.4%)
Missing	32 (2.9%)	7 (2.5%)	25 (3.0%)	8 (2.8%)	9 (3.4%)	8 (2.9%)
Wife spent > 3mos away from village last year						
No	1031 (93.8%)	257 (93.5%)	774 (93.9%)	276 (95.5%)	245 (93.9%)	253 (92.3%)
Yes	61 (5.6%)	14 (5.1%)	47 (5.7%)	13 (4.5%)	15 (5.7%)	19 (6.9%)
Missing	7 (0.6%)	4 (1.5%)	3 (0.4%)	0 (0.0%)	1 (0.4%)	2 (0.7%)
Husband spent > 3mos away from village last year						
No	324 (29.5%)	68 (24.7%)	256 (31.1%)	71 (24.6%)	108 (41.4%)	77 (28.1%)
Yes	738 (67.2%)	198 (72.0%)	540 (65.5%)	210 (72.7%)	143 (54.8%)	187 (68.2%)
Missing	37 (3.4%)	9 (3.3%)	28 (3.4%)	8 (2.8%)	10 (3.8%)	10 (3.6%)
Household assets, mean (SD)	2.1 (1.2)	2.2 (1.1)	2.0 (1.2)	1.9 (1.1)	2.1 (1.2)	2.0 (1.2)

^a^Baseline characteristics were ascertained for N = 27 women missing surveys at baseline who provided surveys at follow-up

Treatment and control arms were not entirely equivalent at baseline. Husbands in the control villages were more likely to have no schooling, to have spent more than 3 months away from the village in the past year, and to have a higher average number of assets than husbands in treatment arms (34% vs 28%, p = 0.01; 72% vs 66%, p < 0.05; and 2.2 vs 2.0, p < 0.01, respectively) (see Table 1).

Adolescent wives were more likely to be lost to follow-up if they were nulliparous at baseline (15% nulliparous, 12% 1 birth, 8% 2 or more births; p = 0.02) or if their husband was polygamous (17% polygamous vs 12% monogamous; p = 0.06; data not shown). There were no differences in female retention rates across other demographics or across study arms.

The primary study outcome, reported current use of modern contraceptives among non-pregnant women, increased substantially over the study period, increasing from 11.8 to 38.3% overall; 17.0% to 29.2% among control participants and from 10.2 to 41.3% among intervention participants (p < 0.01; see Table 2). This change differed by specific intervention arm: modern contraceptive use among non-pregnant women increased from 6.3 to 40.0% in Arm 1, 17.1% to 40.4% in Arm 2, and 8.0% to 43.5% in Arm 3.

**Table 2:** Primary and secondary outcomes at baseline and follow-up, by study arm

	Current modern family planning use, non-pregnant women only				Experience of IPV within past 12 months			
	Baseline n (%)	p-value^a^	Follow-up n (%)	p-value^a^	Baseline n (%)	p-value^a^	Follow-up n (%)	p-value^a^
N (adolescent wives)	933		843		1050		957	
Overall	110 (11.8%)		323 (38.3%)		93 (8.9%)		94 (9.8%)	
Treatment status		0.01		0.002		0.21		0.26
Control	37 (17.0%)		61 (29.2%)		18 (6.9%)		28 (11.7%)	
Intervention	73 (10.2%)		262 (41.3%)		75 (9.5%)		66 (9.2%)	
Study arm		< 0.001		0.01		< 0.001		0.31
Control	37 (17.0%)		61 (29.2%)		18 (6.9%)		28 (11.7%)	
Arm 1—home visits only	16 (6.3%)		88 (40.0%)		11 (3.9%)		23 (9.0%)	
Arm 2—group sessions only	38 (17.1%)		80 (40.4%)		26 (10.6%)		16 (7.2%)	
Arm 3—home visits and group sessions	19 (8.0%)		94 (43.5%)		38 (14.6%)		27 (11.3%)	

^a^Fisher’s exact test

Women participating in the RMA intervention were more than twice as likely to report modern contraceptive use at follow-up relative to those in the control arm (adjusted IRR [aIRR] 2.33, 95% CI 1.41–3.87, p = 0.001; see Table 3). These intervention effects differed by intervention arm. In arm-specific analyses, women were significantly more likely to report modern contraceptive use at follow-up than control in Arms 1 and 3 (aIRR 3.65, 95% CI 1.51–8.78, p = 0.004 and aIRR 2.99, 95% CI 1.68–5.32, p < 0.001, respectively) (see Table 4). Arm 2 participants did not have significantly different likelihood of modern contraceptive use relative to control participants.

**Table 3:** Mixed-effects Poisson regression models assessing the effect of the RMA intervention on current modern family planning use and experience of IPV within the past year, pooled intervention groups

	Current modern family planning use, non-pregnant women only						Experience of IPV within past 12 months					
	Minimally adjusted			Adjusted			Minimally adjusted			Adjusted		
	aIRR	95% CI	p-value	aIRR	95% CI	p-value	aIRR	95% CI	p-value	aIRR	95% CI	p-value
Treatment × time interaction												
Intervention × follow-up	2.26	[1.32, 3.87]	0.003	2.33	[1.41, 3.87]	0.001	0.57	[0.29, 1.13]	0.11	0.57	[0.29, 1.13]	0.11
Treatment status												
Control	Ref.	Ref.	Ref.	Ref.	Ref.	Ref.	Ref.	Ref.	Ref.	Ref.	Ref.	Ref.
Intervention	0.63	[0.36, 1.12]	0.11	0.56	[0.33, 0.96]	0.03	1.39	[0.83, 2.31]	0.21	1.45	[0.86, 2.43]	0.16
Time												
Baseline	Ref.	Ref.	Ref.	Ref.	Ref.	Ref.	Ref.	Ref.	Ref.	Ref.	Ref.	Ref.
Follow-up	1.76	[1.18, 2.63]	0.01	1.71	[1.18, 2.50]	0.005	1.69	[0.97, 2.96]	0.06	1.69	[0.96, 2.95]	0.07
District												
Loga	Ref.	Ref.	Ref.	Ref.	Ref.	Ref.	Ref.	Ref.	Ref.	Ref.	Ref.	Ref.
Doutchi	1.12	[0.76, 1.65]	0.56	1.14	[0.80, 1.63]	0.47	1.47	[1.02, 2.11]	0.04	1.37	[0.90, 2.08]	0.14
Dosso	0.89	[0.61, 1.29]	0.53	0.89	[0.63, 1.26]	0.52	1.81	[1.26, 2.61]	0.001	1.77	[1.23, 2.55]	0.002
Demographics												
Wife age (years)				0.98	[0.91, 1.05]	0.54				0.92	[0.84, 1.01]	0.07
Wife age at marriage (years)				1.04	[0.99, 1.10]	0.13				1.06	[0.95, 1.17]	0.29
Wife education												
Any modern				Ref.	Ref.	Ref.				Ref.	Ref.	Ref.
Quranic only				0.81	[0.60, 1.09]	0.16				1.00	[0.63, 1.58]	0.99
No schooling				0.81	[0.67, 0.98]	0.03				0.86	[0.63, 1.16]	0.32
Husband education												
Any modern				Ref.	Ref.	Ref.				Ref.	Ref.	Ref.
Quranic only				0.77	[0.61, 0.99]	0.04				1.03	[0.69, 1.53]	0.88
No schooling				0.75	[0.60, 0.93]	0.01				0.96	[0.68, 1.36]	0.83
Wife parity												
None				Ref.	Ref.	Ref.				Ref.	Ref.	Ref.
1 birth				1.56	[1.27, 1.92]	< 0.001				0.89	[0.62, 1.28]	0.53
2 or more births				2.60	[1.98, 3.40]	< 0.001				1.40	[0.94, 2.08]	0.10
Husband monogamous or polygamous												
Monogamous				Ref.	Ref.	Ref.				Ref.	Ref.	Ref.
Polygamous				1.15	[0.93, 1.41]	0.19				1.03	[0.74, 1.43]	0.88
Husband spent > 3mos away from village last year												
No				Ref.	Ref.	Ref.				Ref.	Ref.	Ref.
Yes				0.90	[0.75, 1.08]	0.24				1.08	[0.75, 1.55]	0.69
Household assets				0.96	[0.90, 1.03]	0.29				0.90	[0.79, 1.02]	0.10

All models include nested random effects of individual within village

Village ICC for current contraceptive use: 0.0114. Village ICC for past year IPV: 0.087

**Table 4:** Mixed-effects Poisson regression models assessing the effect of the RMA intervention on current modern family planning use and experience of IPV within the past year, by intervention arm

	Current modern family planning use, non-pregnant women only						Experience of IPV within past 12 months					
	Minimally adjusted			Adjusted			Minimally adjusted			Adjusted		
	aIRR	95% CI	p-value	aIRR	95% CI	p-value	aIRR	95% CI	p-value	aIRR	95% CI	p-value
Study arm × time interaction												
Arm 1 × follow-up	3.52	[1.39, 8.91]	0.01	3.65	[1.51, 8.78]	0.004	1.38	[0.48, 3.93]	0.55	1.39	[0.49, 3.95]	0.54
Arm 2 × follow-up	1.32	[0.74, 2.33]	0.34	1.42	[0.84, 2.41]	0.19	0.40	[0.18, 0.89]	0.02	0.40	[0.18, 0.88]	0.02
Arm 3 × follow-up	3.02	[1.68, 5.43]	< 0.001	2.99	[1.68, 5.32]	< 0.001	0.46	[0.21, 1.00]	0.051	0.46	[0.21, 1.01]	0.052
Study arm												
Control	Ref.	Ref.	Ref.	Ref.	Ref.	Ref.	Ref.	Ref.	Ref.	Ref.	Ref.	Ref.
Arm 1—home visits only	0.26	[0.10, 0.71]	0.01	0.20	[0.08, 0.53]	0.001	0.69	[0.34, 1.40]	0.30	0.62	[0.29, 1.33]	0.22
Arm 2—group sessions only	1.03	[0.49, 2.18]	0.93	0.97	[0.50, 1.87]	0.93	1.22	[0.65, 2.30]	0.53	1.39	[0.74, 2.62]	0.31
Arm 3—home visits and group sessions	0.87	[0.44, 1.70]	0.68	0.82	[0.44, 1.52]	0.53	2.28	[1.20, 4.32]	0.01	2.39	[1.31, 4.36]	0.004
Time												
Baseline	Ref.	Ref.	Ref.	Ref.	Ref.	Ref.	Ref.	Ref.	Ref.	Ref.	Ref.	Ref.
Follow-up	1.78	[1.20, 2.63]	0.004	1.73	[1.20, 2.51]	0.003	1.68	[0.96, 2.93]	0.07	1.68	[0.96, 2.92]	0.07
District												
Loga	Ref.	Ref.	Ref.	Ref.	Ref.	Ref.	Ref.	Ref.	Ref.	Ref.	Ref.	Ref.
Doutchi	0.71	[0.31, 1.63]	0.42	0.62	[0.27, 1.42]	0.26	1.53	[1.12, 2.10]	0.01	1.22	[0.81, 1.84]	0.35
Dosso	0.39	[0.18, 0.82]	0.01	0.35	[0.16, 0.74]	0.01	1.14	[0.69, 1.88]	0.61	0.99	[0.65, 1.49]	0.94
Demographics												
Wife age (years)				0.98	[0.91, 1.05]	0.51				0.92	[0.84, 1.01]	0.09
Wife age at marriage (years)				1.04	[0.99, 1.10]	0.15				1.05	[0.96, 1.16]	0.29
Wife education												
Any modern				Ref.	Ref.	Ref.				Ref.	Ref.	Ref.
Quranic only				0.81	[0.60, 1.09]	0.16				1.00	[0.63, 1.58]	1.00
No schooling				0.80	[0.66, 0.98]	0.03				0.84	[0.62, 1.14]	0.27
Husband education												
Any modern				Ref.	Ref.	Ref.				Ref.	Ref.	Ref.
Quranic only				0.76	[0.60, 0.96]	0.02				1.00	[0.68, 1.48]	1.00
No schooling				0.75	[0.60, 0.94]	0.01				0.96	[0.68, 1.35]	0.81
Wife parity												
None				Ref.	Ref.	Ref.				Ref.	Ref.	Ref.
1 birth				1.54	[1.24, 1.90]	< 0.001				0.87	[0.60, 1.24]	0.43
2 or more births				2.62	[2.00, 3.43]	< 0.001				1.36	[0.92, 2.02]	0.13
Husband monogamous or polygamous												
Monogamous				Ref.	Ref.	Ref.				Ref.	Ref.	Ref.
Polygamous				1.17	[0.96, 1.42]	0.13				1.01	[0.73, 1.40]	0.95
Husband spent > 3mos away from village last year												
No				Ref.	Ref.	Ref.				Ref.	Ref.	Ref.
Yes				0.88	[0.73, 1.05]	0.16				1.06	[0.74, 1.53]	0.75
Household assets				0.95	[0.89, 1.02]	0.18				0.90	[0.79, 1.02]	0.09

All models include nested random effects of individual within village

Village ICC for current contraceptive use: 0.006. Village ICC for past year IPV: < 0.001

Post-hoc analyses to examine whether intervention effects on contraceptive use were limited to younger or older participants were consistent with overall models, showing significant associations between intervention and modern contraceptive use in both baseline age groups for Arm 1 (Age 13–16: aIRR 5.57, 95% CI 1.47–21.05, p = 0.01; Age 17–19: aIRR 3.14, 95% CI 1.21–8.15, p = 0.02) and Arm 3 (Age 13–16: aIRR 4.97, 95% CI 1.31–18.90, p = 0.02; Age 17–19: aIRR 2.56, 95% CI 1.34–4.89, p = 0.004) (see Additional file 1: Table S1). No significant association with modern contraceptive use was found for Arm 2 for either age group.

Post-hoc baseline parity-stratified analyses found no significant association between intervention and modern contraceptive use for nulliparous or multiparous women. However, women with one birth at baseline in Arms 1 and 3 had significant increases in modern contraceptive use (aIRR 6.67, 95% CI 1.54–28.85, p = 0.01; aIRR 5.57, 95% CI 1.83–16.96, p = 0.002, respectively) (see Additional file 1: Table S1). Arm 2 participants reported no significant associations with modern contraceptive use irrespective of parity.

Modern contraceptive use findings were robust to IPC weight sensitivity analyses, with direction and strength of associations for the time-by-treatment effects similar to the main regression models (Arm 1 aIRR 4.50, 95% CI 1.71–11.87, p = 0.002; Arm 2 aIRR 1.09, 95% CI 0.54–2.21, p = 0.81; Arm 3 aIRR 2.44, 95% CI 1.20–4.97, p = 0.01) (see Additional file 1: Table S2).

In terms of the secondary outcome, 8.9% of all wives across study arms reported IPV within the past year at baseline, increasing slightly to 9.8% at follow-up (Table 2). This increase was concentrated in the control arm (6.9% at baseline to 11.7% at follow-up), with the intervention arm remaining relatively static (9.5% at baseline to 9.2% at follow-up). This pattern over time differed by specific intervention arm: past year IPV increased from 3.9 to 9.0% in Arm 1, decreased from 10.6 to 7.2% in Arm 2, and decreased from 14.6 to 11.3% in Arm 3.

Women participating in any intervention arm of RMA were slightly less likely to report past year IPV at follow-up relative to those in the control arm, though this difference was not statistically significantly (aIRR 0.57, 95% CI 0.29–1.13, p = 0.11) (see Table 3). In arm-specific analyses, however, adolescent wives in Arm 2 were significantly less likely than those in the control arm to report past year IPV at follow-up (aIRR 0.40, 95% CI 0.18–0.88, p = 0.02) (Table 4). There was a similar magnitude reduction in IPV for married adolescents in Arm 3, though the findings were marginally significant (aIRR 0.46, 95% CI 0.21–1.01, p = 0.052). In contrast, participants in Arm 1 did not report a significant difference in past 12-month IPV relative to controls (aIRR 1.39, 95% CI 0.49–3.95, p = 0.54).

Post-hoc, baseline age-stratified analyses found a significant association between intervention and past year IPV only for wives ages 13–16 years in Arm 2 (aIRR 0.27, 95% CI 0.08–0.88, p = 0.03) (see Additional file 1: Table S3). Although the direction and magnitude of effects on IPV in both Arm 1 and Arm 3 were similar to those seen in the non-stratified analyses, regardless of age (aIRRs 0.36–1.55), no other intervention effects were significant among wives aged 13–16. No significant associations between intervention and IPV were found among wives aged 17–19 years.

Post-hoc, baseline parity-stratified analyses found marginally significant negative associations between intervention and past year IPV for nulliparous women in Arm 2 (aIRR 0.23, 95% CI 0.05–1.05, p = 0.06), and women with 2 or more births at baseline in Arm 3 (aIRR 0.41, 95% CI 0.15–1.13, p = 0.09) (Additional file 1: Table S3). As with age, the direction and magnitude of effects on IPV in Arms 1 and 3 were similar to those seen in the non-stratified analyses; no other significant intervention effects were observed across intervention arm and parity.

Past year IPV findings were robust to IPC weight sensitivity analyses, with direction and strength of associations for the time-by-treatment effects similar to initial adjusted regression models (Arm 1 aIRR 1.63, 95% CI 0.53–4.97, p = 0.39; Arm2 aIRR 0.41, 95% CI 0.17–0.96, p = 0.04; Arm 3 aIRR 0.45, 95% CI 0.20–1.00, p = 0.049) (see Additional file 1: Table S4).

No adverse events were recorded in this study.

## Discussion

RMA, a community-based and gender-synchronized program to promote modern contraceptive use and gender equity, resulted in increased current modern contraceptive use and reduced IPV among married adolescent girls and their husbands in the Dosso region of Niger in this first randomized experimental evaluation of a program of this kind in Francophone West Africa. Overall, RMA participants had a nearly 2.3 factor higher likelihood of current modern contraceptive use after 24 months of intervention, and 0.43 factor lower likelihood of recent IPV.

Only the intervention arm combining both household visits and small group sessions was effective at both increasing current modern contraceptive use and decreasing past year IPV (aIRR = 2.99, p < 0.001 and aIRR = 0.46, p = 0.052, respectively). This suggests that multi-component interventions may be necessary to concurrently shift both contraception and IPV, behaviors deeply influenced by both individual and interpersonal knowledge and behavior, as well as by gender and social norms. This is consistent with other recent experimental evidence underscoring the utility of combined individual and group activities within social and behavior change interventions [22, 23].

Importantly, these improvements were not uniform across different program modalities, and patterns of effects differed for contraceptive and IPV outcomes. The implementation of household visits, with or without small group discussions, appear necessary to achieve significant increases in modern contraceptive use over a 24-month period in this population. This mode of social behavioral intervention focused on increasing knowledge and dispelling misinformation regarding the nature, mechanisms, effects, and potential risks associated with different forms of modern contraception, and how they may be accessed locally. The privacy of these one-on-one, gender-matched visits allows for a dialogue that may result in greater understanding of these topics than from a group setting where the judgement of peers or fear of publicly questioning an authority figure may inhibit questions or dissenting concerns [24].

In contrast, small group discussions were more effective at reducing recent IPV, with or without the addition of household visits. These group discussions, structured to include dialogue between peers, are well suited for examining social norms and their effects on individuals, families and the community as a whole [25, 26]. In this context, gender norms regarding control of fertility and contraceptive use decisions were examined, as well as those regarding the use of force by husbands to maintain control over wives. Since changes in norms require changes in the shared social expectations of any group, small group discussions may be more likely to accomplish such changes relative to individual-level home visits [25, 27]. This opportunity to shift norms may have been further amplified by RMA’s gender-synchronized approach, in which both husbands and wives participated in parallel in these small group discussions [25].

RMA’s observed effects on current modern contraceptive use further differed based on parity of married adolescents, with baseline nulliparous participants not significantly increasing their use of modern contraceptives, in contrast to women with one birth at baseline. As women’s social status in Niger is associated with increased childbearing, childless women may be held in lower social regard [10, 12]. Additionally, the acceptability of nulliparous married girls using contraceptives to prevent their first pregnancy is lower than for women and girls who have children [10, 28]. In this context, community health workers—despite training on equal treatment of all participants—may thus have had a different nature of interaction with nulliparous women (e.g., less information on methods provided, less support for use conveyed). Importantly, this same stigma also affects the behavior of local facility-based health providers [29], very likely further reducing the likelihood of contraceptive access and use among those married adolescents without children.

These results must be interpreted in light of key limitations. All data was collected via respondent self-report, and is therefore subject to recall and social desirability bias, particularly when discussing sensitive topics such as IPV; this was mitigated to the extent possible by asking about current or recent events for the primary and secondary outcomes, and by conducting interviews individually and in private locations. While our treatment and control arms did not have equivalency at baseline in several assessed factors, we adjusted for these factors in multivariable analyses to provide unbiased estimates of treatment effect. Our findings are specific to the population of adolescent wives and their husbands that we studied in Dosso region, Niger; the RMA intervention should be tested elsewhere to assess validity in other contexts.

## Conclusions

In sum, the RMA social behavioral intervention, inclusive of household visits from health workers and gender synchronized small groups for husbands and wives, demonstrated effectiveness in improving modern contraceptive use and reducing victimization from IPV among married adolescent girls in the Dosso region of Niger. Our four-arm design suggests that household visits may be more important for contraceptive use and small groups may be more important for reduction of IPV in this setting, but that a combined intervention approach was optimal for concurrently addressing both contraceptive use and IPV. This pattern suggests that changes in contraceptive use may be reliant on knowledge transmitted by a community expert in a more private setting, while changes in IPV may be reliant on the social norms changes achieved via a group process that allows for shifts in shared expectations among peers. Social norms and stigma may well affect delivery and impact of social behavioral interventions such as RMA, as those married adolescents receiving the program who did not have children, individuals for whom the use of contraceptives is least acceptable, did not increase their contraceptive use. Identifying the modes of social and behavior change programming able to most synergistically and effectively improve contraceptive use and reduce IPV in a setting with the world’s highest fertility and low levels of gender equity is a key step towards improving the health and well-being of these very young adolescent wives.

## Supplementary Information

**Additional file 1: Table S1.** Mixed-effects Poisson regression models assessing the effect of the RMA intervention on current modern family planning use among non-pregnant women. Stratified by baseline age and parity. **Table S2.** Mixed-effects Poisson regression models utilizing IPC weights assessing the effect of the RMA intervention on current modern family planning use among non-pregnant women. **Table S3.** Mixed-effects Poisson regression models assessing the effect of the RMA intervention on past year experiences of IPV. Stratified by baseline age and parity. **Table S4.** Mixed-effects Poisson regression models utilizing IPC weights assessing the effect of the RMA intervention on past year experiences of IPV.

## Data Availability

De-identified data and analysis code are available from the corresponding author upon reasonable request.

## Abbreviations

aIRR: Adjusted incidence rate ratio
CHW: Community health worker
CI: Confidence interval
cRCT: Cluster randomized controlled trial
IPC: Inverse probability of censoring
IPV: Intimate partner violence
IRR: Incidence rate ratio
IUD: Intrauterine device
LAM: Lactational amenorrhea
RMA: Reaching Married Adolescents
SD: Standard deviation
