# Supplementary material for: Effects of the reaching married adolescents program on modern contraceptive use and intimate partner violence: results of a cluster randomized controlled trial among married adolescent girls and their husbands in Dosso, Niger
Source: Reprod Health. 2023 Jun 5;20:83. doi: 10.1186/s12978-023-01609-9 (PMC10243049; doi:10.1186/s12978-023-01609-9)
Supplement: Supplementary file 1 — Additional file 1: Table S1. Mixed-effects Poisson regression models assessing the effect of the RMA intervention on current modern family planning use among non-pregnant women. Stratified by baseline age and parity. Table S2. Mixed-effects Poisson regression models utilizing IPC weights assessing the effect of the RMA intervention on current modern family planning use among non-pregnant women. Table S3. Mixed-effects Poisson regression models assessing the effect of the RMA intervention on past year experiences of IPV. Stratified by baseline age and parity. Table S4. Mixed-effects Poisson regression models utilizing IPC weights assessing the effect of the RMA intervention on past year experiences of IPV. [file 12978_2023_1609_MOESM1_ESM.docx]

**Table** **S1. Mixed-effects Poisson regression models assessing the effect of the RMA intervention on current modern family planning use among non-pregnant women. Stratified by baseline age & parity^1^**

|  | **Overall** | | | **Wife age at baseline** | | | | | | **Wife parity at baseline** | | | | | | | | |
| --- | --- | --- | --- | --- | --- | --- | --- | --- | --- | --- | --- | --- | --- | --- | --- | --- | --- | --- |
|  |  |  |  | **Age 13-16** | | | **Age 17-19** | | | **0** | | | **1** | | | **2+** | | |
|  | aIRR | 95% CI | p-value | aIRR | 95% CI | p-value | aIRR | 95% CI | p-value | aIRR | 95% CI | p-value | aIRR | 95% CI | p-value | aIRR | 95% CI | p-value |
| **Study arm x time interaction** |  |  |  |  |  |  |  |  |  |  |  |  |  |  |  |  |  |  |
| **Arm 1 x follow-up** | 3·52 | [1·39,8·91] | 0·01 | 5·57 | [1·47,21·05] | 0·01 | 3·14 | [1·21,8·15] | 0·02 | - | - | - | 6·67 | [1·54,28·85] | 0·01 | 1·72 | [0·54,5·42] | 0·36 |
| **Arm 2 x follow-up** | 1·32 | [0·74,2·33] | 0·34 | 1·95 | [0·76,5·01] | 0·16 | 1·15 | [0·59,2·22] | 0·69 | 3·34 | [0·29,38·54] | 0·33 | 1·86 | [0·81,4·27] | 0·14 | 0·81 | [0·35,1·88] | 0·63 |
| **Arm 3 x follow-up** | 3·02 | [1·68,5·43] | <0·001 | 4·97 | [1·31,18·90] | 0·02 | 2·56 | [1·34,4·89] | 0·004 | 2·46 | [0·25,23·92] | 0·44 | 5·57 | [1·83,16·96] | 0·002 | 1·56 | [0·67,3·66] | 0·31 |
| Study arm |  |  |  |  |  |  |  |  |  |  |  |  |  |  |  |  |  |  |
| Control | *Ref* | *Ref* | *Ref* | *Ref* | *Ref* | *Ref* | *Ref* | *Ref* | *Ref* | *Ref* | *Ref* | *Ref* | *Ref* | *Ref* | *Ref* | *Ref* | *Ref* | *Ref* |
| Arm 1 - Home visits only | 0·26 | [0·10,0·71] | 0·01 | 0·26 | [0·06,1·16] | 0·08 | 0·29 | [0·11,0·74] | 0·01 | - | - | - | 0·14 | [0·04,0·44] | 0·001 | 0·57 | [0·16,2·02] | 0·38 |
| Arm 2 - Group sessions only | 1·03 | [0·49,2·18] | 0·93 | 0·64 | [0·29,1·43] | 0·28 | 1·16 | [0·50,2·70] | 0·73 | 0·35 | [0·03,3·56] | 0·37 | 1·02 | [0·29,3·59] | 0·97 | 1·54 | [0·83,2·84] | 0·17 |
| Arm 3 - Home visits and group sessions | 0·87 | [0·44,1·70] | 0·68 | 1·78 | [0·20,16·07] | 0·61 | 0·88 | [0·47,1·63] | 0·68 | 1·39 | [0·13,14·86] | 0·79 | 0·47 | [0·14,1·49] | 0·20 | 1·24 | [0·68,2·27] | 0·49 |
| Time |  |  |  |  |  |  |  |  |  |  |  |  |  |  |  |  |  |  |
| Baseline | *Ref* | *Ref* | *Ref* | *Ref* | *Ref* | *Ref* | *Ref* | *Ref* | *Ref* | *Ref* | *Ref* | *Ref* | *Ref* | *Ref* | *Ref* | *Ref* | *Ref* | *Ref* |
| Follow-up | 1·78 | [1·20,2·63] | 0·004 | 1·98 | [1·02,3·86] | 0·05 | 1·78 | [1·13,2·80] | 0·01 | 7·78 | [2·02,30·01] | 0·003 | 1·17 | [0·63,2·17] | 0·62 | 1·90 | [0·97,3·71] | 0·06 |
| District |  |  |  |  |  |  |  |  |  |  |  |  |  |  |  |  |  |  |
| Loga | *Ref* | *Ref* | *Ref* | *Ref* | *Ref* | *Ref* | *Ref* | *Ref* | *Ref* | *Ref* | *Ref* | *Ref* | *Ref* | *Ref* | *Ref* | *Ref* | *Ref* | *Ref* |
| Doutchi | 0·71 | [0·31,1·63] | 0·42 | 1·31 | [0·52,3·35] | 0·57 | 0·71 | [0·31,1·65] | 0·43 | 1·04 | [0·35,3·06] | 0·94 | 0·43 | [0·13,1·43] | 0·17 | 1·03 | [0·43,2·47] | 0·96 |
| Dosso | 0·39 | [0·18,0·82] | 0·01 | 0·23 | [0·03,1·76] | 0·16 | 0·41 | [0·22,0·77] | 0·01 | 0·44 | [0·09,2·00] | 0·29 | 0·31 | [0·14,0·66] | 0·003 | 0·57 | [0·26,1·24] | 0·16 |

^1^All models include nested random effects of individual within village

| **Table S2. Mixed-effects Poisson regression models utilizing IPC weights assessing the effect of the RMA intervention on current modern family planning use among non-pregnant women** | | | |
| --- | --- | --- | --- |
|  |  | **Overall** |  |
|  | aIRR | 95% CI | p-value |
| **Study arm x time interaction** |  |  |  |
| **Arm 1 x Follow-up** | 4.50 | [1.71,11.87] | 0.002 |
| **Arm 2 x Follow-up** | 1.09 | [0.54,2.21] | 0.81 |
| **Arm 3 x Follow-up** | 2.44 | [1.20,4.97] | 0.01 |
| Study arm |  |  |  |
| Control | *Ref* | *Ref* | *Ref* |
| Arm 1 - Home visits only | 0.19 | [0.06,0.55] | 0.002 |
| Arm 2 - Group sessions only | 1.31 | [0.55,3.11] | 0.54 |
| Arm 3 - Home visits and group sessions | 1.22 | [0.56,2.63] | 0.61 |
| Time |  |  |  |
| Baseline | *Ref* | *Ref* | *Ref* |
| Follow-up | 2.09 | [1.32,3.31] | 0.002 |
| District |  |  |  |
| Loga | *Ref* | *Ref* | *Ref* |
| Doutchi | 0.62 | [0.25,1.50] | 0.29 |
| Dosso | 0.32 | [0.14,0.73] | 0.01 |
|  |  |  |  |
| *Models include nested random effects of individual within village and IPC weights | | | |

| **Table** **S3. Mixed-effects Poisson regression models assessing the effect of the RMA intervention on past year experiences of IPV. Stratified by baseline age & parity^1^**   \|  \| **Overall** \| \| \| **Wife age at baseline** \| \| \| \| \| \| **Wife parity at baseline** \| \| \| \| \| \| \| \| \| \| --- \| --- \| --- \| --- \| --- \| --- \| --- \| --- \| --- \| --- \| --- \| --- \| --- \| --- \| --- \| --- \| --- \| --- \| --- \| \|  \|  \|  \|  \| **Age 13-16** \| \| \| **Age 17-19** \| \| \| **0** \| \| \| **1** \| \| \| **2+** \| \| \| \|  \| aIRR \| 95% CI \| p-value \| aIRR \| 95% CI \| p-value \| aIRR \| 95% CI \| p-value \| aIRR \| 95% CI \| p-value \| aIRR \| 95% CI \| p-value \| aIRR \| 95% CI \| p-value \| \| **Study arm x time interaction** \|  \|  \|  \|  \|  \|  \|  \|  \|  \|  \|  \|  \|  \|  \|  \|  \|  \|  \| \| **Arm 1 x Follow-up** \| 1·38 \| [0·48,3·93] \| 0·55 \| 1·55 \| [0·13,17·97] \| 0·73 \| 1·32 \| [0·47,3·67] \| 0·59 \| 2·32 \| [0·39,13·77] \| 0·36 \| 0·67 \| [0·16,2·73] \| 0·57 \| 1·54 \| [0·35,6·73] \| 0·56 \| \| **Arm 2 x Follow-up** \| 0·40 \| [0·18,0·89] \| 0·02 \| 0·27 \| [0·08,0·88] \| 0·03 \| 0·55 \| [0·20,1·51] \| 0·25 \| 0·23 \| [0·05,1·05] \| 0·06 \| 0·73 \| [0·19,2·82] \| 0·65 \| 0·40 \| [0·11,1·39] \| 0·15 \| \| **Arm 3 x Follow-up** \| 0·46 \| [0·21,1·00] \| 0·051 \| 0·36 \| [0·10,1·32] \| 0·12 \| 0·50 \| [0·22,1·16] \| 0·11 \| 0·54 \| [0·13,2·24] \| 0·39 \| 0·44 \| [0·11,1·73] \| 0·24 \| 0·41 \| [0·15,1·13] \| 0·09 \| \| Study arm \|  \|  \|  \|  \|  \|  \|  \|  \|  \|  \|  \|  \|  \|  \|  \|  \|  \|  \| \| Control \| Ref \| Ref \| Ref \| Ref \| Ref \| Ref \| Ref \| Ref \| Ref \| Ref \| Ref \| Ref \| Ref \| Ref \| Ref \| Ref \| Ref \| Ref \| \| Arm 1 - Home visits only \| 0·69 \| [0·34,1·40] \| 0·30 \| 0·30 \| [0·03,2·72] \| 0·28 \| 0·91 \| [0·44,1·87] \| 0·80 \| 0·58 \| [0·15,2·29] \| 0·44 \| 0·92 \| [0·24,3·57] \| 0·91 \| 0·92 \| [0·15,5·49] \| 0·93 \| \| Arm 2 - Group sessions only \| 1·22 \| [0·65,2·30] \| 0·53 \| 1·87 \| [0·91,3·85] \| 0·09 \| 0·90 \| [0·40,2·01] \| 0·80 \| 1·34 \| [0·39,4·68] \| 0·64 \| 1·00 \| [0·27,3·63] \| 0·99 \| 1·45 \| [0·51,4·09] \| 0·48 \| \| Arm 3 - Home visits and group sessions \| 2·28 \| [1·20,4·32] \| 0·01 \| 2·79 \| [0·67,11·67] \| 0·16 \| 2·15 \| [1·25,3·71] \| 0·01 \| 1·91 \| [0·66,5·53] \| 0·23 \| 2·39 \| [0·47,12·03] \| 0·29 \| 2·60 \| [1·12,6·01] \| 0·03 \| \| Time \|  \|  \|  \|  \|  \|  \|  \|  \|  \|  \|  \|  \|  \|  \|  \|  \|  \|  \| \| Baseline \| Ref \| Ref \| Ref \| Ref \| Ref \| Ref \| Ref \| Ref \| Ref \| Ref \| Ref \| Ref \| Ref \| Ref \| Ref \| Ref \| Ref \| Ref \| \| Follow-up \| 1·68 \| [0·96,2·93] \| 0·07 \| 1·50 \| [0·53,4·20] \| 0·44 \| 1·74 \| [0·96,3·15] \| 0·07 \| 1·59 \| [0·56,4·52] \| 0·39 \| 1·86 \| [0·62,5·57] \| 0·27 \| 1·72 \| [0·77,3·86] \| 0·19 \| \| District \|  \|  \|  \|  \|  \|  \|  \|  \|  \|  \|  \|  \|  \|  \|  \|  \|  \|  \| \| Loga \| Ref \| Ref \| Ref \| Ref \| Ref \| Ref \| Ref \| Ref \| Ref \| Ref \| Ref \| Ref \| Ref \| Ref \| Ref \| Ref \| Ref \| Ref \| \| Doutchi \| 1·53 \| [1·12,2·10] \| 0·01 \| 0·93 \| [0·39,2·21] \| 0·87 \| 1·86 \| [1·07,3·22] \| 0·03 \| 1·49 \| [0·59,3·80] \| 0·40 \| 1·05 \| [0·37,3·02] \| 0·92 \| 3·78 \| [0·94,15·24] \| 0·06 \| \| Dosso \| 1·14 \| [0·69,1·88] \| 0·61 \| 0·64 \| [0·14,2·84] \| 0·56 \| 1·37 \| [0·78,2·40] \| 0·27 \| 1·15 \| [0·47,2·80] \| 0·76 \| 0·71 \| [0·14,3·62] \| 0·68 \| 3·00 \| [0·70,12·87] \| 0·14 \|   ^1^All models include nested random effects of individual within village |
| --- | --- | --- | --- | --- | --- | --- | --- | --- | --- | --- | --- | --- | --- | --- | --- | --- | --- | --- | --- | --- | --- | --- | --- | --- | --- | --- | --- | --- | --- | --- | --- | --- | --- | --- | --- | --- | --- | --- | --- | --- | --- | --- | --- | --- | --- | --- | --- | --- | --- | --- | --- | --- | --- | --- | --- | --- | --- | --- | --- | --- | --- | --- | --- | --- | --- | --- | --- | --- | --- | --- | --- | --- | --- | --- | --- | --- | --- | --- | --- | --- | --- | --- | --- | --- | --- | --- | --- | --- | --- | --- | --- | --- | --- | --- | --- | --- | --- | --- | --- | --- | --- | --- | --- | --- | --- | --- | --- | --- | --- | --- | --- | --- | --- | --- | --- | --- | --- | --- | --- | --- | --- | --- | --- | --- | --- | --- | --- | --- | --- | --- | --- | --- | --- | --- | --- | --- | --- | --- | --- | --- | --- | --- | --- | --- | --- | --- | --- | --- | --- | --- | --- | --- | --- | --- | --- | --- | --- | --- | --- | --- | --- | --- | --- | --- | --- | --- | --- | --- | --- | --- | --- | --- | --- | --- | --- | --- | --- | --- | --- | --- | --- | --- | --- | --- | --- | --- | --- | --- | --- | --- | --- | --- | --- | --- | --- | --- | --- | --- | --- | --- | --- | --- | --- | --- | --- | --- | --- | --- | --- | --- | --- | --- | --- | --- | --- | --- | --- | --- | --- | --- | --- | --- | --- | --- | --- | --- | --- | --- | --- | --- | --- | --- | --- | --- | --- | --- | --- | --- | --- | --- | --- | --- | --- | --- | --- | --- | --- | --- | --- | --- | --- | --- | --- | --- | --- | --- | --- | --- | --- | --- | --- | --- | --- | --- | --- | --- | --- | --- | --- | --- | --- | --- | --- | --- | --- | --- | --- | --- | --- | --- | --- | --- | --- | --- | --- | --- | --- | --- | --- | --- | --- | --- | --- | --- | --- | --- | --- | --- | --- | --- | --- | --- | --- | --- | --- | --- | --- | --- | --- | --- | --- | --- | --- | --- | --- | --- | --- | --- | --- | --- | --- | --- | --- | --- | --- | --- | --- | --- | --- | --- | --- | --- | --- | --- | --- | --- | --- | --- | --- | --- | --- | --- | --- | --- | --- | --- | --- | --- | --- | --- | --- | --- | --- | --- | --- | --- | --- | --- | --- | --- | --- |

| **Table S4. Mixed-effects Poisson regression models utilizing IPC weights assessing the effect of the RMA intervention on past year experiences of IPV** | | | |
| --- | --- | --- | --- |
|  |  | **Overall** |  |
|  | aIRR | 95% CI | p-value |
| **Study arm x time interaction** |  |  |  |
| **Arm 1 x Follow-up** | 1.63 | [0.53,4.97] | 0.39 |
| **Arm 2 x Follow-up** | 0.41 | [0.17,0.96] | 0.04 |
| **Arm 3 x Follow-up** | 0.45 | [0.20,1.00] | 0.049 |
| Study arm |  |  |  |
| Control | *Ref* | *Ref* | *Ref* |
| Arm 1 - Home visits only | 0.67 | [0.32,1.39] | 0.28 |
| Arm 2 - Group sessions only | 1.12 | [0.49,2.55] | 0.79 |
| Arm 3 - Home visits and group sessions | 2.10 | [0.98,4.47] | 0.06 |
| Time |  |  |  |
| Baseline | *Ref* | *Ref* | *Ref* |
| Follow-up | 1.95 | [1.11,3.43] | 0.02 |
| District |  |  |  |
| Loga | *Ref* | *Ref* | *Ref* |
| Doutchi | 1.84 | [1.25,2.71] | 0.002 |
| Dosso | 1.46 | [0.80,2.65] | 0.22 |
|  |  |  |  |
| *Models include nested random effects of individual within village and IPC weights | | | |
